# Photodynamic Inactivation of Root Canal Bacteria by Light Activation through Human Dental Hard and Simulated Surrounding Tissue

**DOI:** 10.3389/fmicb.2016.00929

**Published:** 2016-06-15

**Authors:** Fabian Cieplik, Andreas Pummer, Christoph Leibl, Johannes Regensburger, Gottfried Schmalz, Wolfgang Buchalla, Karl-Anton Hiller, Tim Maisch

**Affiliations:** ^1^Department of Conservative Dentistry and Periodontology, University Medical Center RegensburgRegensburg, Germany; ^2^Private PracticeGeiselhöring, Germany; ^3^Department of Dermatology, University Medical Center RegensburgRegensburg, Germany; ^4^Department of Preventive, Restorative and Pediatric Dentistry, School of Dental Medicine, University of BernBern, Switzerland

**Keywords:** photodynamic, *Enterococcus faecalis*, endodontics, optical fiber, transmission

## Abstract

**Introduction:** Photodynamic inactivation of bacteria (PIB) may be a supportive antimicrobial approach for use in endodontics, but sufficient activation of photosensitizers (PS) in root canals is a critical point. Therefore, aim of this study was to evaluate the ability of PS absorbing blue (TMPyP) or red light (Methylene Blue; MB) for light activation through human dental hard and simulated surrounding tissue to inactivate root canal bacteria.

**Methods:** A tooth model was fabricated with a human premolar and two molars in an acrylic resin bloc simulating the optical properties of a porcine jaw. The distal root canal of the first molar was enlarged to insert a glass tube (external diameter 2 mm) containing PS and stationary-phase *Enterococcus faecalis*. Both PS (10 μM) were irradiated for 120 s with BlueV (20 mW/cm^2^; λ_em_ = 400–460 nm) or PDT 1200L (37.8 mW/cm^2^; λ_em_ = 570–680 nm; both: Waldmann Medizintechnik), respectively. Irradiation parameters ensured identical numbers of photons absorbed by each PS. Three setups were chosen: irradiating the glass pipette only (G), the glass pipette inside the single tooth without (GT) and with (GTM) simulated surrounding tissues. Colony forming units (CFU) were evaluated. Transmission measurements of the buccal halves of hemisected mandibular first molars were performed by means of a photospectrometer.

**Results:** PIB with both PS led to reduction by ≥ 5 log_10_ of *E. faecalis* CFU for each setup. From transmission measurements, a threshold wavelength λ_th_ for allowing an amount of light transmission for sufficient activation of PS was determined to be 430 nm.

**Conclusion:** This study can be seen as proof of principle that light activation of given intra-canal PS from outside a tooth may be possible at wavelengths ≥ 430 nm, facilitating clinical application of PIB in endodontics.

## Introduction

It is well established that effective disinfection of the root canal system is crucial for success of endodontic treatment. State-of-the-art procedures involve mechanical debridement and intra-canal irrigation with antimicrobial, tissue dissolving or chelating agents (such as chlorhexidine, sodium hypochlorite or EDTA; Zehnder, 2006). Different medicaments as inter-appointment dressings are used for enhancing disinfection and dissolution of pulpal soft tissue or suppressing inflammation, e.g., based on calcium hydroxide (Mohammadi et al., 2012) or as combined corticosteroid-antibiotic pastes (e.g., Ledermix, Odontopaste; (Athanassiadis et al., 2007). However, most of these substances exhibit evident disadvantages, such as potential development of resistances in bacteria (Arias and Murray, 2009), risk of drug hypersensitivity (Kawashima et al., 2009), or biocompatibility problems (Schmalz, 2014), vague duration of action (Hecker et al., 2013), inactivation by organic compounds (Abouassi et al., 2014), or esthetic limitations due to staining of teeth (Kim et al., 2000). In addition, the presence of resistant pathogens may cause refractory processes of endodontic infections (Al-Ahmad et al., 2014; Łysakowska et al., 2015).

Consequently, alternative approaches as supportive tools for antimicrobial application in endodontics are a current research focus with the photodynamic inactivation of bacteria (PIB) being one option (Gursoy et al., 2013; Chrepa et al., 2014; Cieplik et al., 2014). The bactericidal effect of PIB is based on the excitation of a *per se* non-toxic dye, the so-called photosensitizer (PS), by light of an appropriate wavelength. Upon irradiation, the PS molecule is converted into an excited state from where there are two reaction mechanisms: type I mechanism describes the transfer of charge to a substrate or molecular oxygen resulting in reactive oxygen species (ROS) such as hydroxyl radicals and superoxide ions. Type II mechanism describes the direct transfer of energy to molecular oxygen with generation of the highly reactive singlet oxygen (^1^O_2_;Wainwright, 1998; Schweitzer and Schmidt, 2003).

PIB has already shown promising results *in vitro*, regarding inactivation of planktonic cultures of bacteria (Maisch et al., 2009; Späth et al., 2014) as wells as yeasts (Gonzales et al., 2013) or microorganisms embedded in biofilms (Cieplik et al., 2013, 2015; Gonzales et al., 2013; Voos et al., 2014). However, for bridging the gap between basic and clinical research more realistic *ex vivo* test models are necessary in order to better mimic the situation in the human oral cavity. Here, extracted human teeth are commonly utilized, either by making use of the potentially existing root canal micro flora of those teeth (Ng et al., 2011) or artificially infected after an autoclavation process (Foschi et al., 2007; Fimple et al., 2008).

For example, Ng et al. (2011) studied the effect of PIB on infected human teeth *ex vivo*. Freshly extracted teeth with pulpal necrosis received either conventional chemo-mechanical debridement (CMD) with 6% NaOCl or CMD followed by PIB. Methylene Blue (MB) was used as a PS at a concentration of 50 μg/ml. Root canals were incubated with MB for 5 min, dried with paper points and irradiated for 5 min with a diode laser. This was coupled to a 250-μm diameter optical fiber (output power: 1 W; central wavelength: 665 nm; light dose: 30 J/cm^2^) allowing light distribution at 360° within the root canal. CMD followed by PIB showed a significantly higher efficacy in inactivation of bacteria with 86.5% bacteria-free root canals compared to 49% by CMD alone (Ng et al., 2011).

Fimple et al. (2008) prepared root canal specimens from freshly extracted single-rooted human teeth, autoclaved and incubated them with *Actinomyces israelii, Fusobacterium nucleatum, Porphyromonas gingivalis*, and *Prevotella intermedia* for 3 days. After that period canals were filled with MB (67 μM) as PS and incubated for 10 min. Subsequently, excess MB solution was removed and the canals were irradiated for 5 min (2×2.5 min intermitted by 2.5 min break) with the diode laser as described above. PIB achieved up to 80% reduction of CFU counts (Fimple et al., 2008).

However, all these experimental setups have in common that the PS were activated from inside the root canal. Consequently, Garcez et al. (2013) ascertained the benefit of an optical diffusor for endodontic photodynamic therapy and reasoned the importance of an intra-canal irradiation for appropriate light activation of a given PS. However, to the best of our knowledge, there has not been published any study so far on the activation of a given PS from outside the tooth through human dental hard and surrounding tissues, which would facilitate the handling of light sources for application of PIB in endodontics.

Consequently, the aim of the present study was to evaluate PIB for light activation from outside the tooth through human dental hard and simulated surrounding tissue regarding inactivation of the endodontic key pathogen *Enterococcus faecalis* using PS activated by red light (Methylene Blue) and blue light (TMPyP). For this purpose, an applied *in vitro* tooth model was established to evaluate the antimicrobial photodynamic efficacy of given PS.

## Materials and Methods

### Ethics Statement

All human teeth used in this study were donated after informed consent according to a protocol approved by an appropriate review board at the University of Regensburg.

### Bacterial Culture

*E. faecalis* (ATCC 29212) was grown overnight at 37°C in Brain Heart Infusion broth (BHI; Sigma–Aldrich, St. Louis, MO, USA) on an orbital shaker. After reaching the stationary phase of growth, cultures were harvested by centrifugation (2500 rpm for 5 min; Megafuge 1.0, Heraeus Sepatech, Osterode, Germany), washed with phosphate-buffered saline (PBS; Sigma–Aldrich, St. Louis, MO, USA) and resuspended in PBS yielding an optical density (OD) of 0.06 measured at 600 nm by means of a photospectrometer (SPECORD^®^ 50 PLUS, Analytik Jena, Jena, Germany). These suspensions were used for PIB experiments.

### Photosensitizers and Light Sources

TMPyP [5,10,15,20-tetrakis(1-methyl-4-pyridinium)-porphyrin tetra-(p-toluenesulfonate)] and Methylene Blue [MB; 3,7-bis(dimethylamino)-phenothiazin-5-ium chloride] were used as PS in this study. Both were obtained from a commercial supplier (Sigma–Aldrich, St. Louis, MO, USA; purity > 99%) and were used as received. All PS suspensions were freshly prepared and stored in the dark at 4°C prior to use.

**FIGURE 1:**
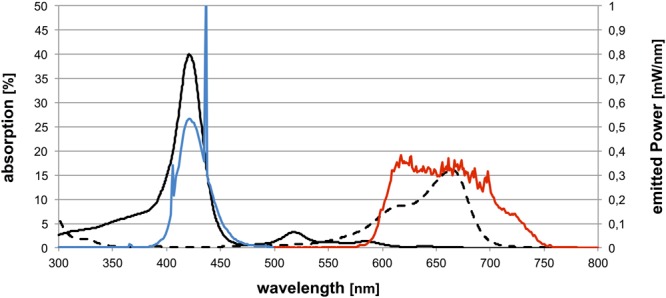
**Characteristic absorption spectra of both PS and emission spectra of corresponding light sources.** The abscissa shows the wavelength in nanometers, the left ordinate the absorption of the PS (%) and the right ordinate the emitted power of the light sources (mW/nm): characteristic absorption spectrum of TMPyP (solid black line) and emission spectrum of Waldmann Blue V (blue line) as well as characteristic absorption spectrum of MB (dashed black line) and emission spectrum of Waldmann PDT 1200L (red line).

The blue light emitting prototype BlueV was used for irradiation of TMPyP, whereas the red light emitting and commercially available light source PDT 1200L (both Waldmann, Villingen-Schwenningen, Germany) was used for irradiation of MB. Characteristic absorption spectra of both PS were measured using a photospectrometer (SPECORD^®^ 50 PLUS, Analytik Jena AG, Jena, Germany). Emission spectra of the corresponding light sources were recorded using a monochromator with CCD detection system (SPEX 232, HORIBA Jobin Yvon, Longjumeau Cedex, France). Spectra are shown in **Figure 1**.

For comparing the biological activity of different PS-light source systems independently from the spectral properties of the respective PS and their corresponding light sources, we calculated identical irradiation parameters by adjusting the numbers of absorbed photons for each PS light source system (Cieplik et al., 2015) to a number of 1.06 × 10^16^ photons per second (TMPyP: BlueV, 20 mW/cm^2^, 120 s, 2.4 J/cm^2^; MB: PDT 1200L, 37.8 mW/cm^2^, 120 s; 4.54 J/cm^2^).

### Tooth Model

**FIGURE 2:**
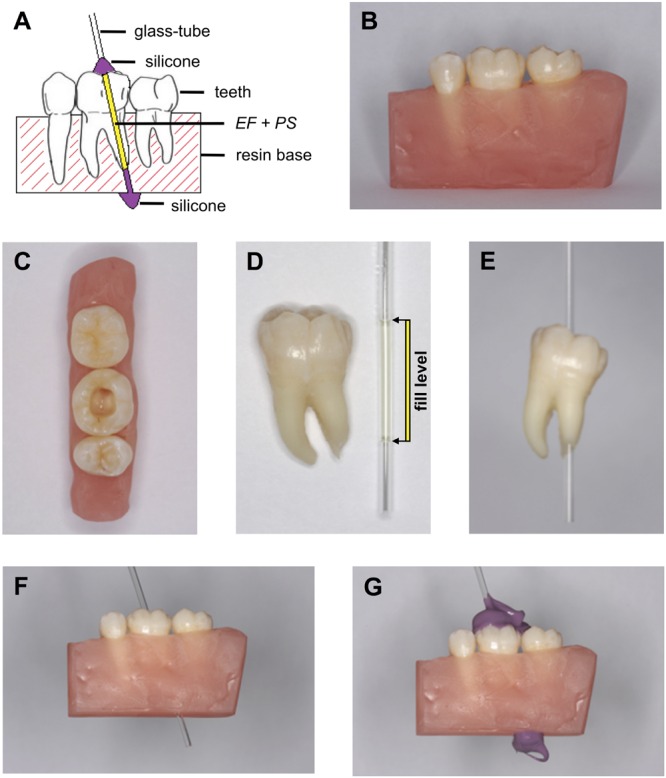
**Tooth model.** Tooth model for evaluation of PIB for light activation through human dental hard and simulated surrounding tissue: **(A)** scheme of the tooth model: a human premolar, first and second molar under proximal contacts in a resin base. The glass tube containing PS + *E. faecalis* is placed in the distal root canal of the first molar and fixed with silicone dental impression material. **(B–F)** Photographic documentation of the tooth model: A human premolar, first molar, and second molar were embedded in a resin base under proximal contacts (**B**). The pulp cavity of the first molar was accessed and the distal root canal was extended (**C**). PS + *E. faecalis* were placed in a glass tube whereby the fill level was adjusted to the tooth length of the first molar (**D**; G setup). The glass tube was placed in the first molar (**E**; GT setup) or in the tooth model (**F**; GTM setup) and fixed with silicone for dental impression (**G**).

The tooth model was fabricated with a human premolar, a first and second human molar (freshly extracted permanent teeth), which were examined visually as well as by radiographs after cleansing to exclude any signs of tooth damage such as caries or fractured cusps. A schematic design of the tooth model is shown in **Figure 2A**.

The teeth were embedded into acrylic resin (Paladur^®^, Heraeus Kulzer, Hanau, Germany) under proximal contacts in order to simulate a mandible lateral teeth area of the third quadrant (**Figure 2B**). A mixture of 76% pink and 24% clear Paladur was used, which has been shown to mimic the transmission of a porcine jaw (buccal resin thickness: 2.9–3.1 mm) (Hiller et al., 2013). All three teeth were removable from the resin base.

The pulp cavity of the first molar was accessed by means of an Endo Access Bur (Dentsply Maillefer, Ballaigues, Suisse, Switzerland) and an airotor (EXPERTtorque E680 L, KaVo, Biberach/Riß, Germany) and its pulp was removed with endodontic files (Pro Taper For Hand Use, Dentsply Maillefer, Ballaigues, Suisse, Switzerland; **Figure 2C**). The distal root canal was instrumented cylindrically to a diameter of 2 mm (Dremel Multi 395, Dremel, Racine, WI, USA) for inserting a glass tube (external diameter: 2 mm; Glass Pasteur Pipette 230 mm length, VWR International, Radnor, PA, USA) containing *E. faecalis* and PS (**Figures 2D,E**). With the same tool, a canal was drilled through the resin bloc to accommodate for the glass-tube extending the root apex (**Figure 2F**). The glass tube was fixed in the single tooth or the tooth model with silicone for dental impression (Silagum-Mono; DMG Chemisch-Pharmazeutische Fabrik, Hamburg, Germany; **Figure 2G**). The bottom and the top part of the glass tube were covered by the silicone, too. In addition, a shadow mask made of black cardboard was used for the experiments in order to ensure that light for activation of the PS must have penetrated through the tooth model instead of being refracted by or transmitted through the top and the bottom of the glass tube.

### Photodynamic Inactivation of Bacteria

About 10 μl of *E. faecalis* suspension was mixed with either 10 μl TMPyP (10 μM) or MB (10 μM) in 96-well microtiter plates (Corning Costar^®^, Corning, NY, USA). This suspension (20 μl) was transferred into a glass tube obtaining final PS concentrations of 10 μM (**Figure 2D**), whereby the fill level was adjusted to the tooth length of the first molar. Fixing of the glass tube in the tooth or the tooth model was done with silicone for dental impression (Silagum-Mono).

Three different experimental setups were chosen: irradiating the glass tube containing *E. faecalis* and PS only (G; **Figure 2D**), the glass tube placed in the single first molar (GT; **Figure 2E**) or the whole tooth model (GTM; **Figure 2G**). Irradiation was for 120 s from the buccal side in all cases (using PDT 1200L for MB and BlueV for TMPyP; **Figure 2F**). Controls were neither sensitized with PS nor irradiated (PS–L–) or were incubated with the PS only (PS+L–) or were exposed to light only (PS–L+). After irradiation, the bacterial suspension was completely removed from the glass tube. Serial tenfold dilutions (10^-2^–10^-7^) were prepared in BHI broth and aliquots (3 × 20 μL) were plated on Mueller–Hinton agar plates (provided by the Institute of Medical Microbiology and Hygiene, University Medical Center Regensburg, Regensburg, Germany), as described earlier (Miles et al., 1938). Plates were incubated aerobically for 24 h at 37°C. Later, colony forming units (CFUs) were counted.

### Measurement of Transmission through Dental Hard Tissue

Five human mandibular first molars were chosen after visual and radiographic inspection in order to exclude any signs of tooth damage such as caries or fractured cusps in order to determine the transmission for wavelengths from 200 to 800 nm. Teeth were thoroughly cleansed and bisected in mesio-distal direction under water cooling by means of a rotating diamond saw (Wild-Heerbrugg, Aarau, Switzerland). While the oral half was discarded, the buccal one was kept in *aqua dest.* after removing any remaining pulpal tissue. Transmission was measured by means of a photospectrometer (SPECORD^®^ 50 PLUS, Analytik Jena, Jena, Germany) with the light path directed from the buccal side toward the pulp chamber at a level ensuring transmission through enamel as well as dentine (diameter 3–4 mm).

### Data Treatment

All PIB results are shown as medians, including 25 and 75% quartiles, which were calculated using SPSS for Windows, version 20 (SPSS Inc., Chicago, IL, USA), from the values of at least six independent experiments. Horizontal solid and dashed lines in PIB figures represent reductions of 3 and 5 log_10_ steps CFU, respectively, compared to untreated control groups (PS–L–). Medians on or below these lines represent PIB efficacy rates of 99.9% (3 log_10_) or 99.999% (5 log_10_) at least, which is declared as biologically relevant antimicrobial activity or disinfectant effect, respectively, according to infection control guidelines (Boyce and Pittet, 2002).

For transmission measurements (data from the buccal halves of five mandibular first molars), transmission was plotted versus wavelength. The resulting dose-response shaped curves were fitted to the best fit as calculated by TableCurve 2D (Systat Software Inc., San Jose, CA, USA). This fit was analyzed for the second derivative maximum, the point of maximal curvature, which was defined as threshold wavelength λ_th_.

## Results

### Photodynamic Inactivation of Bacteria

**FIGURE 3:**
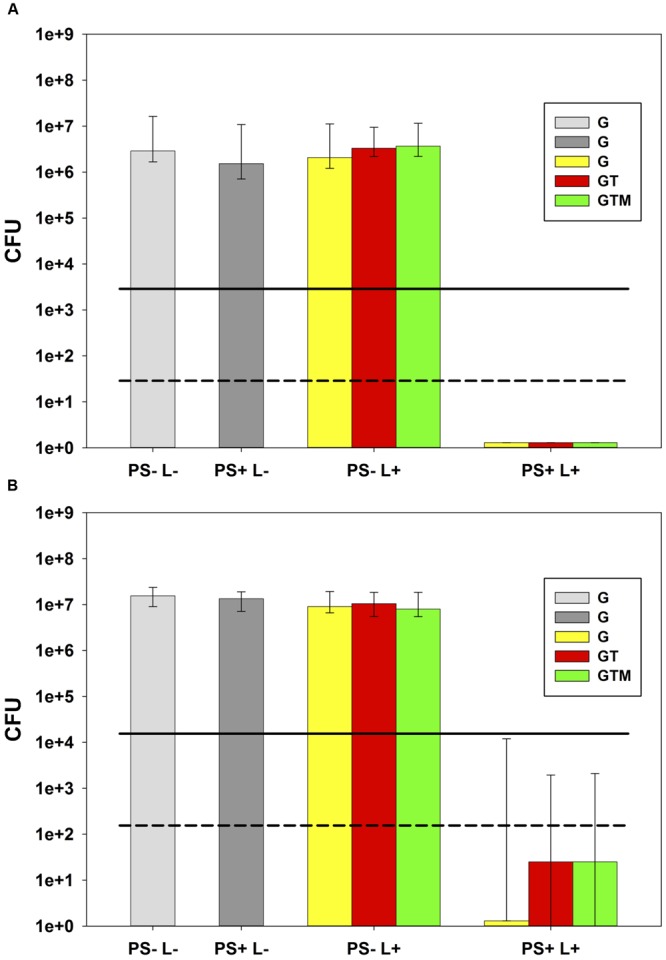
**Photodynamic inactivation of bacteria (PIB).** Photodynamic inactivation of bacteria with both PS-light source systems against *E. faecalis* with light activation in all three setups of the tooth model: glass tube only (G), glass tube inserted in the single tooth (GT) or the whole tooth model (GTM). All PIB experiments in this figure are shown as Colony forming unit (CFU) medians with 25 and 75% quantiles (*n* = 6). Solid and dashed lines depict CFU reductions of ≥ 3 log_10_ or ≥ 5 log_10_ steps, respectively, related to untreated control groups PS–L–. In all cases, there was no CFU-reduction with PS (PS+L–) or light alone (PS–L+). **(A)** PIB with TMPyP and Blue V. PS+L+ groups show inactivation by 6.5 log_10_ below the detection limit regardless which set-up (G; GT; GTM) was applied. **(B)** PIB with MB and PDT 1200L. PS+L+ groups show inactivation by 7.2 log_10_ below the detection limit for irradiation of the glass tube only (G) and by 5.8 log_10_ for irradiation of the single tooth (GT) or the tooth model (GTM).

PIB with TMPyP inactivated *E. faecalis* by 6.5 log_10_ as compared to corresponding untreated control PS–L–, regardless whether the glass tube only (G) was irradiated or the glass tube inserted in the single tooth (GT) or the whole tooth model (GTM; **Figure 3A**). PIB with MB led to inactivation of 7.2 log_10_ as compared to corresponding untreated control PS–L– for irradiation in the glass tube only (G), whereas irradiation of the glass tube inside the single tooth (GT) or incorporated in the whole tooth model (GTM) revealed inactivation efficacy of 5.8 log_10_ (**Figure 3B**). Treatment with PS only (PS+L–) or light only (PS–L+) had no effect on CFU of *E. faecalis*.

### Tooth Transmission Measurements

**FIGURE 4:**
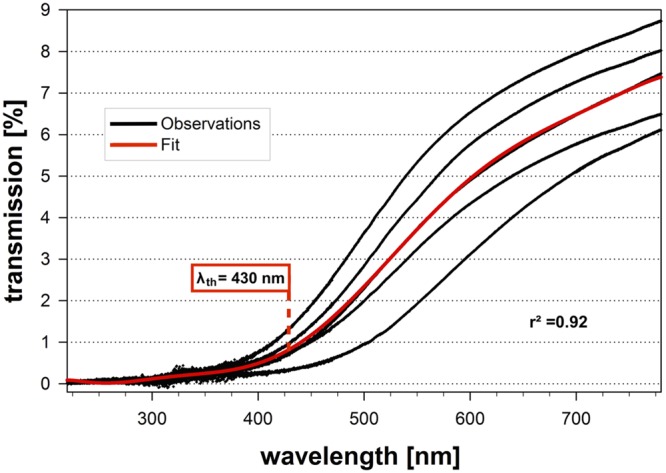
**Transmission measurements.** Observed transmission measurements of the buccal halves of five human mandibular first molars in the range 200–800 nm (black dots). Red line depicts resulting four-parameter sigmoidal fit with *r*^2^ = 0.92 as calculated by TableCurve 2D. 95% confidence intervals are depicted, but are overlayed by the line representing the best fit. The threshold wavelength λ_th_ (second derivative maximum, the point of maximal curvature) was determined to be λ_th_ = 430 nm exhibiting 0.84% transmission at this point.

The best fit of the transmission values of the buccal halves of five human mandibular first molars as calculated by TableCurve 2D resulted in a four-parameter sigmoidal curve exhibiting *r*^2^ = 0.92 (**Figure 4**). The threshold wavelength λ_th_ (second derivative maximum, the point of maximal curvature) was determined to be λ_th_ = 430 nm exhibiting 0.84% transmission at this point.

## Discussion

The aim of this study was to evaluate the efficacy of PIB for light activation from outside the tooth through human dental hard and simulated surrounding tissue in a model approach for avoiding problems of intra-canal placement of the activating light source. For this purpose, a tooth model has been fabricated. The composition of the resin used for embedding the teeth was based on data from transmission measurements of a porcine mandible and comparing them to different formulations of acrylic resins (Hiller et al., 2013). Therefore, it may mimic the alveolar bone and gingival tissue around the teeth for the purpose of this study although the lack of blood flow has to be considered as a drawback. The teeth used for the model were thoroughly cleansed and stored moistly; autoclaving of the teeth was waived since autoclaving might have changed the photophysical properties of enamel and dentine and might have led to desiccation. The glass of the glass tube containing PS and *E. faecalis* caused a reduction of light transmission of approximately 10% (as specified by the manufacturer). However, using the glass tube was necessary, though, for being able to entirely retrieve the applied amount of PS and *E. faecalis* after PIB without any dilution effects caused by incomplete recovery. Furthermore, due to the glass tube, loss of bacteria or of PS by diffusion into dentinal tubules could also be excluded, by which reproducibility is improved. In this experimental setup, porphyrin derivative TMPyP and phenothiazinium derivative MB were used as PS since porphyrin and phenothiazinium dyes are widely used as PS in dental and dermatological clinical practice and for investigating whether there is a difference in efficacy between extra-dental activation by blue (TMPyP) and red light (MB) due to optical effects.

The light sources used in this study were chosen, as their emission spectra cover the absorption maxima of the respective PS. For ensuring reliable comparison of both PS-light source systems, the spectral emission of both light sources and the absorption spectra of both PS as well as the distribution of the emitted photons have to be considered and the numbers of photons absorbed by distinct PS have to be adjusted (Cieplik et al., 2015). In this way, irradiation of 10 μM TMPyP with BlueV for 120 s at 20 mW/cm^2^ (light dose 4.54 J/cm^2^) equals irradiation of 10 μM MB with PDT 1200L for 120 s at 37.8 mW/cm^2^ (light dose: 2.4 J/cm^2^) with a number of 1.06 × 10^16^ photons absorbed per second for each PS-light source system.

The Gram-positive, facultative aerobe *E. faecalis* was used as a model organism in this study since it is strongly associated with different forms of periradicular disease, primary endodontic infections as well as persistent infections after failed endodontic treatment (Stuart et al., 2006). A major characteristic of *E. faecalis* is its ability to withstand very high alkaline conditions (pH 11.5; Evans et al., 2002; McHugh et al., 2004), which elucidates the importance of alternative or supportive regimens like PIB for treatment of refractory endodontic infections.

Here, TMPyP as well as MB exhibited disinfecting efficacy with blue light activated TMPyP even being slightly more effective (bacterial reduction of 6.5 log_10_ steps) than MB (5.8 log_10_ steps) when light activated through dental tissues in the single tooth (GT) or the tooth model (GTM). Furthermore, for both PS there was virtually no difference between irradiating both PS in the glass tube only (G) or inserted in the single tooth (GT) or in the whole tooth model (GTM).

It is known that human teeth are able to transmit (red) light to the pulp, whereby enamel as well as dentine acts as light guides, albeit with anisotropic optical scattering (Odor et al., 1996; Kienle et al., 2002, 2006; Kienle and Hibst, 2006). When light hits on enamel prisms, which are arranged perpendicular to the surface, it is transmitted to the amelo-dentinal junction and guided toward the pulp along dentinal tubules (Odor et al., 1996; Kienle and Hibst, 2006). Hereby, light propagation in dentine is due to multiple scattering caused by the cylindrical microstructure, but not by total reflection as in optical fibers (Kienle et al., 2002, 2006; Kienle and Hibst, 2006), since optical fibers consist of a core with high refraction index and a cladding with low refraction-index, whereas in dentine the refraction index of the tubules is lower than of peritubular dentine (Kienle and Hibst, 2006). Light propagation in enamel is similar due to the prisms building a structure of aligned cylindrical scatterers (Kienle et al., 2006).

However, the similar efficacy rates for PS activation by blue and red light seem unexpected since it is well known that the penetration of light through tissue is dependent on its wavelength, with light from longer wavelengths penetrating tissue to a greater extent than light from shorter wavelengths (Hirmer et al., 2012). Therefore, we did transmission measurements of bisected human first mandibular molars and measured the transmission through their buccal halves. The resulting dose–response curves were fitted and the fit was analyzed for the point of maximal curvature (second derivative maximum), which was defined as threshold wavelength λ_th_ for the shortest wavelength that penetrates dental tissue proper enough for activating a given PS molecule and was found to be 430 nm. Although the transmission at this wavelength was 0.84% only, activation of TMPyP at the tested concentration of 10 μM was sufficient in the GTM setup for achieving a disinfecting efficacy. This may be explained due to an overlap of its Soret-band with this threshold wavelength. Furthermore, the measured transmission values likely underestimate the actual transmission through the tooth halves due to scattering effects in the light path inside the photospectrometer. Consequently, it may be possible to activate PS from outside the tooth through human dental hard and simulated surrounding tissue may be possible for wavelengths longer than 430 nm. Activating an intra-canal PS from outside the tooth may facilitate the use of PIB in endodontics, since, there are commonly known limitations for optical fibers, e.g., intense bendings of the root canal or the risk of a fracture of the optical fiber inside the root canal, therefore complicating endodontic application of PIB.

It may be a limitation of this study is that the efficacy of PIB was evaluated against planktonic cells, whereas in infected root canals bacteria are situated in a sessile biofilm mode. However, as this study was thought to be a proof of principle of extra-dental irradiation for PIB, we used planktonic bacteria due to their more simple culture and their greater sensitivity to most antimicrobial approaches for being able to measure small differences in antimicrobial efficacy. As this proof of principle was successful, in the next step the model has to be modified in order to investigate whether biofilms may also be inactivated by PIB with light activation through dental hard and simulated surrounding tissue.

## Conclusion

The tooth model presented in this study allows evaluation of the antimicrobial photodynamic efficacy of given PS for light activation through human dental hard and simulated surrounding tissue. Under these conditions, the tested PS activated by either blue (TMPyP) or red light (Methylene Blue) resulted in inactivation rates of more than 5 log_10_ steps reduction of CFU, respectively. Transmission measurements of bisected human molars resulted in a threshold wavelength λ_th_ for allowing an amount of light transmission for appropriate activation of a PS was 430 nm. Therefore, it may be possible to activate PS absorbing at wavelength ranges above this threshold from outside the tooth. However, before transferring these results to the clinical situation there should be undertaken further tests on biofilms.

## Author Contributions

KAH, TM, FC, and GS conceived and designed the experiments. AP, CL, and JR performed the experiments. KAH, FC, TM, JR, AP, WB, and GS analyzed the data. FC, AP, TM, KAH, CL, GS, and WB wrote the paper.

## Conflict of Interest Statement

The authors declare that the research was conducted in the absence of any commercial or financial relationships that could be construed as a potential conflict of interest.

The reviewer TMBR and handling Editor declared their shared affiliation and the handling Editor states that the process nevertheless met the standards of a fair and objective review.

## References

[B1] Abouassi T. Hannig C. Mahncke K. Karygianni L. Wolkewitz M. Hellwig E. (2014). Does human saliva decrease the antimicrobial activity of chlorhexidine against oral bacteria? *BMC Res. Notes* 7:711 10.1186/1756-0500-7-711 PMC4200226 25300308

[B2] Al-Ahmad A. Ameen H. Pelz K. Karygianni L. Wittmer A. Anderson A. C. (2014). Antibiotic resistance and capacity for biofilm formation of different bacteria isolated from endodontic infections associated with root-filled teeth. *J. Endod.* 40 223–230. 10.1016/j.joen.2013.07.023 24461408

[B3] Arias C. A. Murray B. E. (2009). Antibiotic-resistant bugs in the 21st century–a clinical super-challenge. *N. Engl. J. Med.* 360 439–443. 10.1056/NEJMp0804651 19179312

[B4] Athanassiadis B. Abbott P. V. Walsh L. J. (2007). The use of calcium hydroxide, antibiotics and biocides as antimicrobial medicaments in endodontics. *Aust. Dent. J.* 52 S64–S82. 10.1111/j.1834-7819.2007.tb00527.x 17546863

[B5] Boyce J. M. Pittet D. (2002). Guideline for hand hygiene in health-care settings: recommendations of the healthcare infection control practices advisory committee and the HICPAC/SHEA/APIC/IDSA hand hygiene task force. *Infect. Control Hosp. Epidemiol.* 23 3–40. 10.1086/503164 12515399

[B6] Chrepa V. Kotsakis G. A. Pagonis T. C. Hargreaves K. M. (2014). The effect of photodynamic therapy in root canal disinfection: a systematic review. *J. Endod.* 40 891–898. 10.1016/j.joen.2014.03.005 24935531

[B7] Cieplik F. Pummer A. Regensburger J. Hiller K.-A. Späth A. Tabenski L. (2015). The impact of absorbed photons on antimicrobial photodynamic efficacy. *Front. Microbiol.* 6:706 10.3389/fmicb.2015.00706 PMC4502582 26236292

[B8] Cieplik F. Späth A. Regensburger J. Gollmer A. Tabenski L. Hiller K.-A. (2013). Photodynamic biofilm inactivation by SAPYR-An exclusive singlet oxygen photosensitizer. *Free Radic. Biol. Med.* 65 477–487. 10.1016/j.freeradbiomed.2013.07.031 23891675

[B9] Cieplik F. Tabenski L. Buchalla W. Maisch T. (2014). Antimicrobial photodynamic therapy for inactivation of biofilms formed by oral key pathogens. *Front. Microbiol.* 5:405 10.3389/fmicb.2014.00405 PMC4130309 25161649

[B10] Evans M. Davies J. K. Sundqvist G. Figdor D. (2002). Mechanisms involved in the resistance of *Enterococcus faecalis* to calcium hydroxide. *Int. Endod. J.* 35 221–228. 10.1046/j.1365-2591.2002.00504.x 11985673

[B11] Fimple J. L. Fontana C. R. Foschi F. Ruggiero K. Song X. Pagonis T. C. (2008). Photodynamic treatment of endodontic polymicrobial infection in vitro. *J. Endod.* 34 728–734. 10.1016/j.joen.2008.03.011 18498901 PMC2596687

[B12] Foschi F. Fontana C. R. Ruggiero K. Riahi R. Vera A. Doukas A. G. (2007). Photodynamic inactivation of *Enterococcus faecalis* in dental root canals in vitro. *Lasers Surg. Med.* 39 782–787. 10.1002/lsm.20579 18081066

[B13] Garcez A. S. Fregnani E. R. Rodriguez H. M. Núñez S. C. Sabino C. P. Suzuki H. (2013). The use of optical fiber in endodontic photodynamic therapy. Is it really relevant? *Lasers Med. Sci.* 28 79–85. 10.1007/s10103-012-1073-8 22399242

[B14] Gonzales F. P. Felgenträger A. Bäumler W. Maisch T. (2013). Fungicidal photodynamic effect of a twofold positively charged porphyrin against *Candida albicans* planktonic cells and biofilms. *Future Microbiol.* 8 785–797. 10.2217/fmb.13.44 23701333

[B15] Gursoy H. Ozcakir-Tomruk C. Tanalp J. Yilmaz S. (2013). Photodynamic therapy in dentistry: a literature review. *Clin. Oral Investig.* 17 1113–1125. 10.1007/s00784-012-0845-7 23015026

[B16] Hecker S. Hiller K.-A. Galler K. M. Erb S. Mader T. Schmalz G. (2013). Establishment of an optimized ex vivo system for artificial root canal infection evaluated by use of sodium hypochlorite and the photodynamic therapy. *Int. Endod. J.* 46 449–457. 10.1111/iej.12010 23240861

[B17] Hiller K.-A. Christa T. Niklas A. Danilov S. N. Ganichev S. D. Schmalz G. (2013). An in-vitro-model of a human jaw for testing optical properties. *J. Dent. Res.* 92 3716.

[B18] Hirmer M. Danilov S. N. Giglberger S. Putzger J. Niklas A. Jäger A. (2012). Spectroscopic Study of Human Teeth and Blood from Visible to Terahertz Frequencies for Clinical Diagnosis of Dental Pulp Vitality. *J Infrared Milli. Terahz. Waves* 33 366–375. 10.1007/s10762-012-9872-3

[B19] Kawashima N. Wadachi R. Suda H. Yeng T. Parashos P. (2009). Root canal medicaments. *Int. Dent. J.* 59 5–11. 19323305

[B20] Kienle A. Forster F. K. Diebolder R. Hibst R. (2002). Light propagation in dentin: influence of microstructure on anisotropy. *Phys. Med. Biol.* 48 N7–N14. 10.1088/0031-9155/48/2/401 12587909

[B21] Kienle A. Hibst R. (2006). Light guiding in biological tissue due to scattering. *Phys. Rev. Lett.* 97 018104 10.1103/PhysRevLett.97.018104 16907413

[B22] Kienle A. Michels R. Hibst R. (2006). Magnification–a new look at a long-known optical property of dentin. *J. Dent. Res.* 85 955–959. 10.1177/154405910608501017 16998140

[B23] Kim S. T. Abbott P. V. McGinley P. (2000). The effects of Ledermix paste on discolouration of mature teeth. *Int. Endod. J.* 33 227–232. 10.1046/j.1365-2591.2000.00278.x 11307439

[B24] Łysakowska M. E. Ciebiada-Adamiec A. Sienkiewicz M. Sokołowski J. Banaszek K. (2015). The cultivable microbiota of primary and secondary infected root canals, their susceptibility to antibiotics and association with the signs and symptoms of infection. *Int. Endod. J.* 49 422–430. 10.1111/iej.12469 26011084

[B25] Maisch T. Wagner J. Papastamou V. Nerl H. J. Hiller K.-A. Szeimies R.-M. (2009). Combination of 10% EDTA, Photosan, and a blue light hand-held photopolymerizer to inactivate leading oral bacteria in dentistry in vitro. *J. Appl. Microbiol.* 107 1569–1578. 10.1111/j.1365-2672.2009.04342.x 19457024

[B26] McHugh C. P. Zhang P. Michalek S. Eleazer P. D. (2004). pH required to kill *Enterococcus faecalis* in vitro. *J. Endod.* 30 218–219. 10.1097/00004770-200404000-00008 15085049

[B27] Miles A. A. Misra S. S. Irwin J. O. (1938). The estimation of the bactericidal power of the blood. *J. Hyg. (Lond.)* 38 732–749. 10.1017/S002217240001158X 20475467 PMC2199673

[B28] Mohammadi Z. Shalavi S. Yazdizadeh M. (2012). Antimicrobial activity of calcium hydroxide in endodontics: a review. *Chonnam Med. J.* 48 133–140. 10.4068/cmj.2012.48.3.133 23323217 PMC3539092

[B29] Ng R. Singh F. Papamanou D. A. Song X. Patel C. Holewa C. (2011). Endodontic photodynamic therapy ex vivo. *J. Endod.* 37 217–222. 10.1016/j.joen.2010.10.008 21238805 PMC3034089

[B30] Odor T. M. Watson T. F. Pitt Ford T. R. McDonald F. (1996). Pattern of transmission of laser light in teeth. *Int. Endod. J.* 29 228–234. 10.1111/j.1365-2591.1996.tb01374.x 9206438

[B31] Schmalz G. (2014). Strategies to improve biocompatibility of dental materials. *Curr. Oral Health Rep.* 1 222–231. 10.1007/s40496-014-0028-5

[B32] Schweitzer C. Schmidt R. (2003). Physical mechanisms of generation and deactivation of singlet oxygen. *Chem. Rev.* 103 1685–1757. 10.1021/cr010371d 12744692

[B33] Späth A. Leibl C. Cieplik F. Lehner K. Regensburger J. Hiller K.-A. (2014). Improving photodynamic inactivation of bacteria in dentistry: highly effective and fast killing of oral key pathogens with novel tooth-colored type-ii photosensitizers. *J. Med. Chem.* 57 5157–5168. 10.1021/jm4019492 24884918

[B34] Stuart C. H. Schwartz S. A. Beeson T. J. Owatz C. B. (2006). *Enterococcus faecalis*: its role in root canal treatment failure and current concepts in retreatment. *J. Endod.* 32 93–98. 10.1016/j.joen.2005.10.049 16427453

[B35] Voos A. C. Kranz S. Tonndorf Martini S. Voelpel A. Sigusch H. Staudte H. (2014). Photodynamic antimicrobial effect of safranine O on an ex vivo periodontal biofilm. *Lasers Surg. Med.* 46 235–243. 10.1002/lsm.22217 24473989

[B36] Wainwright M. (1998). Photodynamic antimicrobial chemotherapy (PACT). *J. Antimicrob. Chemother.* 42 13–28. 10.1093/jac/42.1.13 9700525

[B37] Zehnder M. (2006). Root canal irrigants. *J. Endod.* 32 389–398. 10.1016/j.joen.2005.09.014 16631834

